# Neural Entrainment to Rhythmically Presented Auditory, Visual, and Audio-Visual Speech in Children

**DOI:** 10.3389/fpsyg.2012.00216

**Published:** 2012-07-19

**Authors:** Alan James Power, Natasha Mead, Lisa Barnes, Usha Goswami

**Affiliations:** ^1^Department of Experimental Psychology, Centre for Neuroscience in Education, University of CambridgeCambridge, UK

**Keywords:** entrainment, audio-visual speech perception, rhythm

## Abstract

Auditory cortical oscillations have been proposed to play an important role in speech perception. It is suggested that the brain may take temporal “samples” of information from the speech stream at different rates, phase resetting ongoing oscillations so that they are aligned with similar frequency bands in the input (“phase locking”). Information from these frequency bands is then bound together for speech perception. To date, there are no explorations of neural phase locking and entrainment to speech input in children. However, it is clear from studies of language acquisition that infants use both visual speech information and auditory speech information in learning. In order to study neural entrainment to speech in typically developing children, we use a rhythmic entrainment paradigm (underlying 2 Hz or delta rate) based on repetition of the syllable “ba,” presented in either the auditory modality alone, the visual modality alone, or as auditory-visual speech (via a “talking head”). To ensure attention to the task, children aged 13 years were asked to press a button as fast as possible when the “ba” stimulus violated the rhythm for each stream type. Rhythmic violation depended on delaying the occurrence of a “ba” in the isochronous stream. Neural entrainment was demonstrated for all stream types, and individual differences in standardized measures of language processing were related to auditory entrainment at the theta rate. Further, there was significant modulation of the preferred phase of auditory entrainment in the theta band when visual speech cues were present, indicating cross-modal phase resetting. The rhythmic entrainment paradigm developed here offers a method for exploring individual differences in oscillatory phase locking during development. In particular, a method for assessing neural entrainment and cross-modal phase resetting would be useful for exploring developmental learning difficulties thought to involve temporal sampling, such as dyslexia.

## Introduction

The acquisition of language is a fundamental human achievement of great cognitive importance. Language acquisition is achieved by the majority of children without obvious difficulties, even by children with cognitive developmental impairments such as Down syndrome (Dodd and Crosbie, 2011). Language acquisition follows a protracted time course, with notable individual differences (Fenson et al., 1994), yet the learning mechanisms underlying speech perception are on-line from birth, providing sensory information about speech that is both auditory and visual (Kuhl, 2004). In fact, the fetus receives both auditory and kinesthetic information about speech from at least the third trimester. The infant is able to hear the mother’s voice through the amniotic fluid (which acts as a low pass filter, hence emphasizing the rhythmic structure of speech). Once the infant is born and can also watch the mother or other carers talk, then visual information about speech such as lip movements and cheek/jaw movements supplements this auditory information with perfect temporal congruity (see Lewkowicz and Ghazanfar, 2011, for a recent summary). The central role of temporal AV congruence in learning may suggest a mechanistic role for oscillatory mechanisms in both the auditory and visual modalities in explaining individual differences in learning about speech, as well as a role for cross-modal phase resetting.

Behaviorally, there is ample evidence demonstrating that multisensory congruency provides a basis for speech processing from birth onward. For example, infants as young as 4 months will become fussy and distressed if shown a “talking head” where the visual speech information is “out of sync” with the auditory information, so that “visual prosody” (the rhythmic facial and head movements that convey information about the speech envelope) and auditory prosody are incongruent (Dodd, 1979). Infants of 4 months also show automatic audio-visual speech integration, displaying the “McGurk effect.” Burnham and Dodd (2004) showed babies one syllable visually (a head silently mouthing [ga]) while presenting a second syllable auditorally [ba], resulting in automatic perception of the intermediate syllable [da]. Infants also show perceptual “narrowing” or neural specialization to the speech sounds and visual speech information that is relevant for the language that they are learning. Whereas babies younger than around 10 months can discriminate phonetic changes used in all languages, thereby showing sensitivity to phoneme boundaries that are not utilized in their own language, they cease responding to distinctions between non-native speech sounds by around 12 months (e.g., Werker and Tees, 1984). Similarly, while younger babies respond to differences in the silent mouthing of syllables in non-native languages (e.g., Spanish babies will discriminate silent mouthing of/ba/versus/va/, even though this is not a phonetic distinction used in Spanish), by around 1 year there has been perceptual narrowing. Spanish-learning infants then cease to discriminate the silent mouthing of these syllables, whereas English-learning infants retain the discrimination (Pons et al., 2009). These and other data suggest that the multisensory coherence of AV information is critical for language acquisition (Lewkowicz et al., 2010), raising the possibility that atypical functioning of the neural mechanisms that support sensitivity to temporal congruence may lead to the development of atypical speech-based representations (Goswami, 2011).

Neural mechanisms for speech perception must function at multiple timescales concurrently, and cortical oscillations may provide a relevant mechanism. Both auditory and visual information in speech unfolds over multiple time scales. Considering auditory structure first, recent advances in our understanding of speech processing have highlighted the critical role played by low frequency modulations in the signal and the amplitude envelope in speech intelligibility (Drullman et al., 1994a,b; Shannon et al., 1995). The amplitude envelope is the variation in the intensity of the sounds made by different articulators, and is dominated by low frequency modulations (4–6 Hz), reflecting its underlying syllabic structure (i.e., opening and closing of the vocal tract). If the envelope is smeared and flattened, intelligibility reduces dramatically, and experiments have shown that most of the important information for intelligibility is in envelope components between 1 and 16 Hz (Drullman et al., 1994a,b). Also important is the rapidly changing spectral content (fine structure), which determines linguistic content (such as the formant transitions underpinning the perceptual change from “ba” to “da”). Hence the speech stream contains modulations at multiple temporal rates, which may convey information about different linguistic aspects of speech such as phonetic segments (approximate rate of 20–50 Hz), syllables (3–7 Hz), and stressed syllables or prosodic information (0.5–3 Hz, see Greenberg et al., 2003; Poeppel, 2003; Drullman, 2006). The functional role of cortical oscillations in speech perception is captured by multi-time resolution models of speech processing proposed on the basis of adult data (Poeppel, 2003; Luo and Poeppel, 2007; Poeppel et al., 2008; Ghitza and Greenberg, 2009; Ghitza, 2011).

According to multi-time resolution models, speech information at multiple timescales is processed and integrated in a hierarchical and interdependent manner via neural phase alignment or neural “entrainment” of oscillatory networks. The auditory system appears to synchronize the ongoing oscillations of large ensembles of neurons to the modulation rates in the stimulus, realigning the phase of neural activity so that peaks in excitability co-occur with peaks in amplitude modulation (Zion Golumbic et al., 2012). Within these multi-time resolution frameworks, conceptual significance is given to entrainment within the theta band (temporal sampling at the “syllable rate”) and the gamma band (temporal sampling at the “phonetic rate”). For example, Luo and Poeppel (2007) demonstrated using MEG that the phase pattern of the theta band (∼4–7 Hz) tracked the amplitude envelope of spoken sentences, thereby segmenting the incoming speech signal into syllable-sized packets or samples of information and resetting and sliding to track speech dynamics. By oscillatory accounts, neural encoding of the amplitude envelope depends on the phase of oscillatory networks resetting to align with “edges” or “onsets” in the signal (Giraud and Poeppel, 2012), perhaps using amplitude rise times (Goswami, 2011). Converging evidence for oscillatory entrainment comes from MEG and EEG non-speech studies employing the auditory steady-state response (ASSR), a power (amplitude) increase in neuronal response at the stimulating rate thought to indicate the synchronization of excitability fluctuations to the amplitude modulation rate of the stimulus (e.g., Picton et al., 1987; Liégeois-Chauvel et al., 2004).

Physiological studies in animal models offer more direct evidence for the phase resetting of cortical oscillations in the encoding of (non-speech) auditory input. For example, the temporal pattern of local field potentials (LFPs) is phase-locked to the temporal structure of complex sounds in non-human primates, particularly in frequency ranges of 2–9 and 16–30 Hz (Kayser et al., 2009; Chandrasekaran et al., 2010). Indeed, Kayser et al. (2009) were able to show experimentally that neuronal spiking (power increases) and LFP phase (entrainment or realignment) below 30 Hz carried complementary information, hence maximizing the information received by the brain about the temporal structure of the signal. The phase of the underlying neuronal oscillations that generate the LFP may hence act to control the timing of neuronal excitability and to gate spiking activity so that it occurs at the most relevant times (e.g., Lakatos et al., 2005; Schroeder and Lakatos, 2009). On this account, neuronal oscillations would provide the temporal context for processing complex stimuli, directing the more intricate processing of the acoustic content to particular points in time (Buzsáki and Chrobak, 1995; Zion Golumbic et al., 2012). Regarding the development of language acquisition, therefore, neural phase locking or auditory entrainment could be critical to the temporal encoding and parsing of the complex speech signal, particularly in the theta (syllable rate) and gamma (phonetic rate) bands. Further, individual differences in auditory entrainment could be one factor in the atypical development of language processing skills found in developmental dyslexia and specific language impairment (SLI, see Goswami, 2011).

Nevertheless, given the importance of visual speech information for speech perception, visual entrainment should also play a crucial role in the mechanisms that underpin the development of speech representations. Most visual speech information is recovered from the lower spatial and temporal frequencies (below seven cycles per face, and six to nine frames per second, respectively), suggestive of the developmental importance of congruity with low frequency auditory modulations (e.g., Munhall et al., 2004). Recent work with adults has for example highlighted the importance of low frequency visual speech cues such as jaw movements for temporal congruency in AV speech processing (Chandrasekaran et al., 2009), finding a close temporal correspondence between the area of the mouth opening and the wideband acoustic envelope. Further, this temporal co-ordination had a distinct rhythm that was between 2 and 7 Hz (the theta band). Drawing on recent oscillatory frameworks for speech perception (e.g., Hickok and Poeppel, 2007; Ghitza and Greenberg, 2009; Ghitza, 2011), Chandrasekaran et al. (2009) observed that the natural temporal features of AV speech signals were optimally structured for the neural (oscillatory) rhythms of the brains of their receivers. Indeed, Schroeder et al. (2008) suggest that the facilitation of speech perception by visual information may be due to neuronal oscillations in auditory areas being modulated by the visual input, so that the related auditory input arrives during a high excitability phase and is thus amplified. Given that visual articulatory cues can precede vocalizations by 200 ms or more, Schroeder et al. (2008) suggest that the visual gestures reset auditory cortex to the “optimal state” for processing the succeeding vocalizations. Developmentally, therefore, it is possible that inefficient *visual* entrainment could affect the phase resetting of auditory cortex for speech input in developmental disorders such as dyslexia and SLI. For receivers such as infants and young children, visual oscillatory entrainment, auditory oscillatory entrainment, and cross-modal AV phase interactions should all be important in supporting the efficient development of speech-based representations in the mental lexicon of word forms.

The potential utility of cross-modal phase resetting for understanding human speech processing is shown by a recent MEG study by Luo et al. (2010). They demonstrated that human auditory cortex tracks both auditory and visual stimulus dynamics using low frequency neuronal phase modulation. Using congruent and incongruent audio-visual stimulation and a phase coherence analysis technique, Luo et al. (2010) were able to measure how the phase of cortical responses was coupled to aspects of stimulus dynamics. They found that the auditory cortex reflected low frequency aspects of the visual signal as well as tracking auditory stimulus dynamics. The visual cortex reflected low frequency aspects of the auditory signal as well as tracking visual stimulus dynamics. Luo et al. identified cross-modal phase modulation as an important neural mechanism underpinning AV integration. Their data showed that the visual stream in a matched movie modulated the auditory cortical activity by aligning the phase to the optimal phase angle, so that the expected auditory input arrived during a high excitability state, consistent with Schroeder et al. (2008). Nevertheless, studies that have measured phase alignment in humans directly are rare, and none (to our knowledge) have measured phase alignment in children.

Accordingly, we present here the first study of phase locking to speech in typically developing children aged 13 years, assessing phase locking to rhythmic auditory, visual, and auditory-visual (AV) stimulus streams [presentation of the syllable “ba” at a 2-Hz (delta band) rate]. Following a period of entrainment, participants were asked to press a button if they heard (auditory condition), saw (visual condition), or both heard and saw (AV condition) a “ba” stimulus that violated the entrained isochronous rhythm. We measured oscillatory entrainment in each condition, and assessed the influence of visual cues on auditory oscillations by comparing the entrainment of the auditory system to rhythmic speech when presented as auditory-alone and when accompanied by natural congruent articulatory facial gestures. Our aim was to establish a paradigm which could be used in future studies of children with developmental language disorders, capable of measuring hypothesized atypical auditory versus atypical visual entrainment to speech, or atypical cross-modal phase resetting.

## Materials and Methods

### Participants

We studied 23 typically developing children with a mean age of 163 months who were taking part in a longitudinal behavioral study of auditory processing (Goswami et al., 2011). All participants and their guardians gave informed consent for EEG in accordance with the Declaration of Helsinki, and the study was approved by the Psychology Research Ethics Committee of the University of Cambridge. All participants were free of any diagnosed learning difficulties (dyspraxia, ADHD, autistic spectrum disorder, speech, and language impairments) and spoke English as their first language.

#### Standardized tests of reading, non-word reading, vocabulary, and IQ

Psychometric tests were given for the purposes of exploring possible relations between entrainment and the development of spoken and written language skills. The psychometric tests comprised the British Ability Scales (BAS; single word reading, Elliott et al., 1996); the phonemic decoding efficiency (PDE) measure of non-word reading from the TOWRE (Torgesen et al., 1999); the British Picture Vocabulary Scales (BPVS receptive vocabulary, Dunn et al., 1982); and one subtest of the Wechsler Intelligence Scale for Children (WISC-III, Wechsler, 1992): picture arrangement. Performance is shown in Table 1. Full-scale IQ scores had been collected earlier in the longitudinal study, and are also shown in Table 1.

**Table 1:** **Participant characteristics**.

	Mean	(SD)
Age in months	163	15
Reading BAS SS	110.3	12.3
Reading age in months	176.5	20.1
BPVS SS	110.5	15.8
Non-word TOWRE SS	107.6	11.3
*WISC Pic Arr*	14.6	3.5
*WISC FSIQ*	116.1	16.8
Phoneme deletion %	83.5	15
PSTM %	64.3	15
RAN (secs)	35.7	4.8

#### Experimental phonological tasks

In order to see whether individual differences in entrainment would relate to individual differences in phonological processing between children, participants were administered a phoneme deletion task, an experimental measure of phonological short-term memory (PSTM) and an experimental measure of rapid automatized naming (RAN). The first two tasks used digitized speech created from a native female speaker of standard Southern British English. In the phoneme deletion task, the children listened to non-word stimuli and were asked to delete a target sound, e.g., “Please say ‘starp’ without the ‘*p*’.” The sounds to be deleted were either initial, medial, or final phonemes, and in each case the deletion resulted in a real word. This was an adaptation of a task used by Pasquini et al. (2007) for adults with dyslexia. The task comprised 20 trials. In the PSTM task children were asked to recall sets of four monosyllabic non-words, e.g., “sool, juff, teed, goak” in the correct order. There were 20 trials in total. Performance was scored in terms of the number of items recalled correctly (total = 80). In the RAN task, children were asked to name line drawings of two sets of familiar objects (first set: *cat*, *shell*, *knob*, *zip*, *thumb*; second set: *web*, *fish*, *book*, *dog*, *cup*; see Richardson et al., 2004). For each set, children were first introduced to the names of the pictures and then shown a page with the same pictures repeated 40 times in random order. The children were asked to produce the names as quickly as possible. Average naming speed across the two lists was then scored. Table 1 shows performance in the experimental tasks (percent correct for phoneme deletion and PSTM, naming time in seconds for RAN).

#### Rhythmic entrainment task

**Figure 1:**
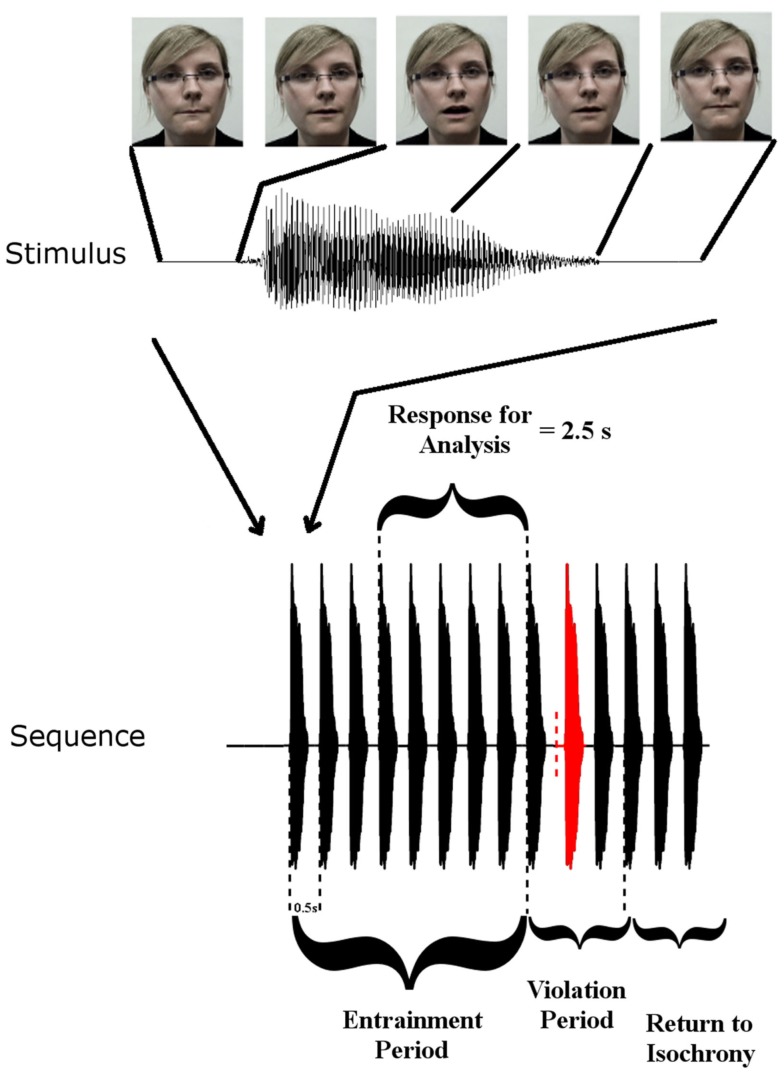
**Stimulus Setup: top panel shows one auditory “Ba” token and corresponding frames of the visual stimulus at five time points**. Visual movement initiates 68 ms before auditory onset. The lower panel shows a stimulus sequence consisting of the entrainment period with SOA of 500 ms, and the violation period where SOA is disrupted, followed by three re-entrainment stimuli (“Return to Isochrony”). The red stimulus is the violator, whose position in the violation period is chosen at random (i.e., either the first, second, or third stimulus can violate the rhythm). The vertical dashed red line indicates where the stimulus would have onset if it had adhered to the isochronous stimulation rate. Also shown is the “Response for Analysis” period over which EEG responses were analyzed.

Rhythmic speech comprising multiple repetitions of the syllable “BA” was presented at a uniform repetition rate of 2 Hz. There were three conditions: auditory (A), visual (V), and auditory-visual (AV). The visual condition involved a silent “talking head” providing natural visual articulatory cues for “BA.” The auditory task comprised the audio track only, and the AV task provided both auditory and visual speech information via a “talking head.” Visual cues initiated 68 ms before auditory “ba” onset. The conditions were blocked such that only one type of stimulation was presented in each block. Each block contained 30 stimulus sequences. Twenty-five of these sequences contained a rhythmic violation from the uniform 2 Hz. The other five sequences did not contain a violation and occurred randomly as catch trials. Each individual stimulus sequence in a block of 30 contained 14 stimuli (see Figure 1). The first eight stimuli in a sequence are hereafter called the entrainment period, where the stimulation rate is always 2 Hz. The following three stimuli are hereafter called the rhythmic violation period. In the rhythmic violation period, one of the three stimuli is randomly selected to be out of time with the 2-Hz beat (hence the violation is not predictable). In order to ensure attention to the rhythmic stream, the child was instructed to respond by way of a button press when this violation was perceived. The remaining three stimuli in the sequence always adhered to the isochronous 2 Hz stimulation rate (“return to isochrony” period). Throughout the auditory-only condition a fixation cross was present on the screen in front of the participants and they were instructed to fix their gaze on it. In the other two conditions participants were instructed to concentrate their gaze on the lips of the “talking head.” Feedback was given after each sequence. Each participant was presented with three blocks of each condition, hence each subject received 90 sequences per condition overall (i.e., 75 target sequences plus 15 catch sequences). The degree to which the violator was out of sync varied depending on how well the participant was doing in the task. If a child correctly identified three violators in three consecutive sequences, then the deviation from 500 ms SOA was reduced by 16.67 ms. If a violator was not detected, then the deviation increased by 16.67 ms. This three-down one-up staircase procedure was employed in an attempt to equate engagement across subjects. The calibration also ensured equal performance accuracy regardless of ability. Theoretically the three-down one-up staircase should result in a performance accuracy of 79.4% for each subject (Levitt, 1971).

### EEG pre-processing

EEG data were recorded and digitized with a 24-bit analog-to-digital (A/D) converter using the 65-channel EGI geodesic Sensor Net system. The sampling rate was 500 Hz. Data pre-processing was done in MATLAB (MathWorks). The data was low pass filtered at 200 Hz prior to A/D conversion to prevent aliasing due to the presence of frequencies above the Nyquist frequency (half the sampling rate, i.e., 250 Hz). The data were then high pass filtered offline at 0.75 Hz and low pass filtered at 45 Hz. High pass filtering the data at 0.75 Hz enabled investigation of activity low enough to assess the expected 2 Hz entrainment in our experimental paradigm. FASTER (Nolan et al., 2010) was used for artifact rejection. FASTER uses statistical thresholds to identify bad channels and interpolates them. It also carries out independent component analysis (ICA) to identify the constituent activations comprising the overall EEG. Components arising from eye blinks and other noise sources such as electrode pop-off (an electrode losing connection on a particular trial) are identified as bad and removed. The data is then returned from component space to the electrode space thus eliminating the artifactual activity.

### EEG analysis

**Figure 2:**
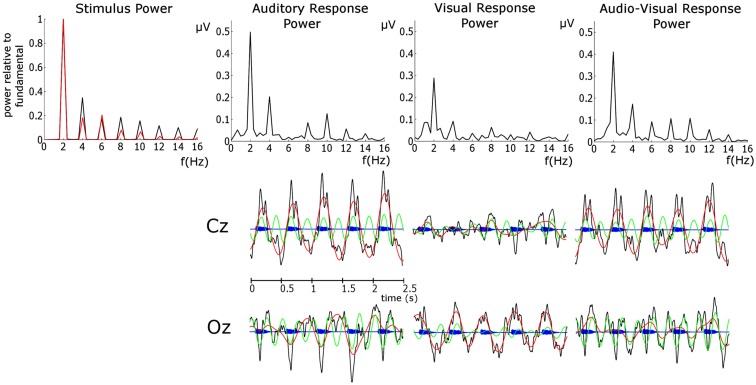
**Upper Panel: FFT spectra of the stimulus and EEG phase-locked response power**. For the stimulus power the auditory response is presented in black and the visual response in red. Here the stimulus power spectrum is normalized by the power at the fundamental in order to assess the relative importance of harmonics. This was achieved by dividing the power in both spectra by the power at the relevant fundamental. Auditory stimulus power was obtained from the stimulus envelope in the entrainment period. Visual stimulus power was obtained from the area between the lips of the talking head (i.e., extent of mouth openness) in the same entrainment period. Lower panel: EEG time courses of the unfiltered response (black), delta response (red), theta response (green) overlain on the auditory stimulus time course (blue).

For all analyzes, the first three observations in each entrainment period were discarded to ensure that rhythmicity had been established (following the approach employed in Gomez-Ramirez et al., 2011). Here we are interested in entrainment to a uniform stimulus repetition rate, and so responses in the violation and “return to isochrony” periods (see Figure 1) were not analyzed. Furthermore, sequences in which a target was not detected were discarded, as were catch trials. As accuracy was ∼79 and 75% target sequences were presented per condition, the analysis included ∼60 trials per subject. In order to identify frequency bands of interest we examined the phase-locked power (i.e., the power of sequence averages in the time period of interest) in the three conditions. To do this, the Fast-Fourier transform (FFT) spectrum of the averaged responses for each condition were obtained. The FFTs were carried out on the responses in the entrainment period resulting in a frequency resolution of 0.4 Hz. Before FFT analysis activity was baseline corrected using the mean activity in the analysis window. Given the peaks evident in the spectra (see Figure 2), with the highest phase-locked power present for delta and theta, we deemed the delta (∼2 Hz) and theta (∼4 Hz) frequency bands to be of interest (for further details see *Results* and *Discussion*). Frequency band activity was obtained using FIR filters designed using the Parks–McClellan algorithm (Parks and McClellan, 1972). The delta band filter had corner frequencies of 1 and 3 Hz and the theta band filter had corner frequencies of 3 and 5 Hz. Both had a 40-dB attenuation in the stop band. In order to examine whether auditory entrainment differed for the A and AV conditions, we subtracted an estimate of phase-locked visual activity from each AV trial (AV–V), and compared the remaining A and (AV–V) activity. The estimate of visual activity was obtained from the time-locked average activity in the visual condition.

### Assessing phase locking

**Figure 3:**
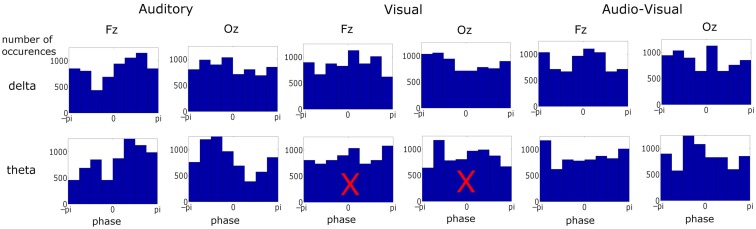
**Phase distribution at visual stimulus onset at representative frontal and occipital electrodes in each condition and frequency band over ∼6900 observations in each plot**. Most distributions differed from uniformity when tested against the Rayleigh statistic at a critical *p*-value of 0.001. Distributions with a superimposed *X* did not result in significant entrainment. There was no significant entrainment for theta band activity in the visual condition.

**Figure 4:**
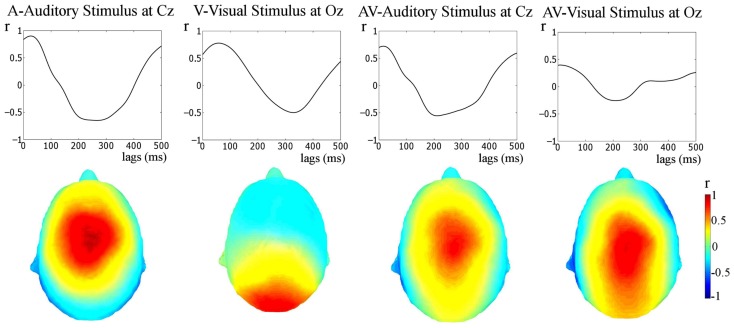
**Stimulus-response cross-correlation analysis**. The upper panel shows the results of the cross-correlation analysis between the stimulus and the responses. The lower panel shows the topographies of Pearson’s *r*-values at the lags of peak correlation. A very strong representation of the temporally extended stimulus is seen in the response.

In each correctly identified target sequence the pre-stimulus phase (at visual stimulus onset) of the last five stimuli in the entrainment period were obtained. These phase values were pooled across sequences and subjects, giving ∼6900 (60 sequences × 5 stimuli × 23 participants) observations, and pre-stimulus phase distribution histograms for each condition were obtained (see Figure 3). The phase values were extracted by obtaining the *analytic signal* of the filtered responses via the Hilbert transform. The *analytic signal* is complex, i.e., it has real and imaginary components, and thus the instantaneous phase can be extracted. To test if pre-stimulus phase distributions differed from uniformity, the distributions for the three conditions were tested against the null hypothesis of uniformity using the Rayleigh statistic at two representative electrodes (Fz and Oz). Given the number of trials obtained per subject (∼60) and the fact that EEG phase measures are highly susceptible to noise we did not consider ∼60 trials adequate to obtain an accurate distribution of phase for each *individual* subject. Therefore, a fixed-effects analysis was carried out and not a random-effects analysis. A critical *p*-value of 0.001 was selected to minimize type I error. Statistical difference from uniformity suggests a preferred concentration of phase values, which is indicative of entrainment (Stefanics et al., 2010; Gomez-Ramirez et al., 2011). In order to obtain converging evidence for entrainment, the relationship between the stimuli and the responses was also assessed using cross-correlations (see Figure 4). We also sought to relate measures of neural entrainment to the behavioral data. To do this we employed inter-trial coherence (ITC; see Table 3; Figure 6). ITC is a measure of phase alignment and can have values ranging from 0 to 1. One indicates perfect phase alignment and 0 indicates no phase alignment. ITC was calculated for the same pre-stimulus phase values that were submitted to the Rayleigh test.

We also sought to investigate the impact of the accompanying visual stimulation on auditory entrainment. The pre-stimulus phase values (at auditory stimulus onset) for the AV and (AV–V) responses were extracted in the same manner as outlined above for the separate conditions. We then looked at the topography of the strength of entrainment. To do this we plotted the pooled phase values at each electrode (see Figure 7, upper panels). These topographies show a common region of high entrainment indicative of entrainment in auditory areas (see Figure 7, lower panels). Subsequent analysis was thus confined to the pooled activity of electrodes in this region of interest (ROI). The electrodes chosen for this ROI are shown in Figure 7. We compared the extent of phase alignment as obtained using ITC, the total power in the frequency bands and the preferred pre-stimulus phase. Total power was determined by computing the FFT for the entrainment period of interest (see Figure 1) for the response to each sequence in each condition, and then averaging the resulting frequency spectra and extracting the power at delta and theta. An estimate of the preferred phase was determined for each subject by finding the mean pre-stimulus phase.

## Results

### Behavioral entrainment task

As shown in Table 2, the visual entrainment task required larger violations for correct detection of 80% of deviants than the auditory or AV tasks. However, the visual entrainment task also showed the fastest reaction times. In order to assess whether there were significant differences between conditions, two one-way ANOVAs by condition were run, one taking threshold in ms as the dependent variable and the other taking RT in ms. The ANOVA for threshold indeed showed a main effect of condition, *F*(2,21) = 76.3, *p* < 0001, η*p*^2^ = 0.879. *Post hoc* inspection of the means (Tukeys) showed that the threshold for the visual condition was significantly higher than the thresholds for the auditory and AV conditions, which did not differ. The ANOVA for reaction time also showed a main effect of condition, *F*(2,21) = 43.6, *p* < 0001, η*p*^2^ = 0.806. *Post hoc* inspection of the means (Tukeys) showed that the reaction time in the visual condition was significantly lower than the reaction times for the auditory and AV conditions, which did not differ.

**Table 2:** **Mean thresholds in milliseconds and reaction time (RT) in milliseconds by condition**.

	Mean	SD
Auditory task (threshold)	3.3	1.3
Visual task (threshold)	6.9	1.8
AV task (threshold)	3.5	1.1
Auditory task (RT)	358.3	47.7
Visual task (RT)	305.5	48.0
AV task (RT)	341.4	42.4

### EEG results

#### Analysis of individual conditions (A, V, and AV)

Figure 2 shows that the dominant stimulus frequency is 2 Hz for both the auditory and visual stimuli, as expected. The auditory stimulus power spectrum was obtained from the stimulus sequence envelope using an FFT. A time domain representation of the visual stimulus was obtained by calculating the area between the lips of the talking head during each video frame (i.e., when closed there is zero area between the lips and when fully open the area is at maximum). The visual stimulus power spectrum was obtained from this temporal-area representation using an FFT. In general, Figure 2 shows that the power at the harmonics is stronger, relative to the fundamental, for the auditory than the visual stimulus. The two most powerful components for the auditory stimulus are the fundamental (2 Hz) and the first harmonic (4 Hz), while the fundamental is the sole dominant frequency for the visual stimulus. Unsurprisingly, we see the dominant response frequency is 2 Hz in all conditions. Furthermore, the response power closely mirrors the stimulus, with 2 and 4 Hz dominant for the auditory condition but 2 Hz alone dominant for the visual condition. The response in the AV condition shows a similar pattern to that of the auditory condition. Given the dominance of the fundamental and the first harmonic in both stimulus and response, we limit further analysis to these two frequencies, and refer to them as delta and theta activity respectively, as per the standard declinations of EEG activity (Buzsáki and Draguhn, 2004). The activity at 2 Hz would be expected given that this is also the frequency of stimulation (Gomez-Ramirez et al., 2011). Theta band activity would also be expected given the frequency content of the stimuli and as theta is argued to play an integral role in speech processing and parsing (Luo and Poeppel, 2007).

We next assessed the pre-stimulus phase distributions of the delta and theta activity (see Figure 3). Once again two representative electrodes were chosen: Fz and Oz, to identify responses which should be predominantly auditory and visual respectively. Significant differences from a uniform random distribution were investigated using the Rayleigh statistic. A critical *p*-value of 0.001 was chosen in order to minimize Type I errors. For the auditory and audio-visual conditions the pre-stimulus phase distribution differed from uniform for both delta (Fz: A_δ_: *p* < 0.0001, *Z* = 173.37; AV_δ_: *p* < 0.001, *Z* = 31.09. Oz: A_δ_: *p* < 0.001, *Z* = 52.10; AV_δ_: *p* < 0.001, *Z* = 36.16) and theta (Fz: A_θ_: *p* < 0.0001, *Z* = 198.47; AV_θ_: *p* < 0.001, *Z* = 28.12. Oz: A_θ_: *p* < 0.0001, *Z* = 277.38; AV_θ_: *p* < 0.001, *Z* = 51.81). However in the visual condition only the delta phase differed from uniform (Fz: *V*_δ_: *p* < 0.001, *Z* = 38.86. Oz: *V*_δ_: *p* < 0.0001, *Z* = 103.62) while the theta distribution did not differ (Fz: *V*_θ_: *p* > 0.01, *Z* = 3.20. Oz: *V*_θ_: *p* > 0.01, *Z* = 4.00). Again, this pattern mirrors the relative importance of these frequency bands in the stimulus. Furthermore, from Figure 3 we can see that the significant Rayleigh statistic in the auditory and visual conditions is driven by concentration of phase around preferred phases. The distributions in the audio-visual condition are somewhat more complicated and in some cases seem to have two phase concentrations (e.g., delta activity at Fz). This is likely due to a number of factors influencing activity in this region. As can be seen from the similarity of the delta time course activity in the auditory and audio-visual conditions (see Figure 2, Lower Panel), while the activity in these conditions is auditory-dominated, it is also influenced by the visual stimulus, both in terms of altering the preferred phase of auditory entrainment (see below) and also the volume conduction of visual sensory activity. This may account for the multi-peaked distribution patterns observed.

**Figure 5:**
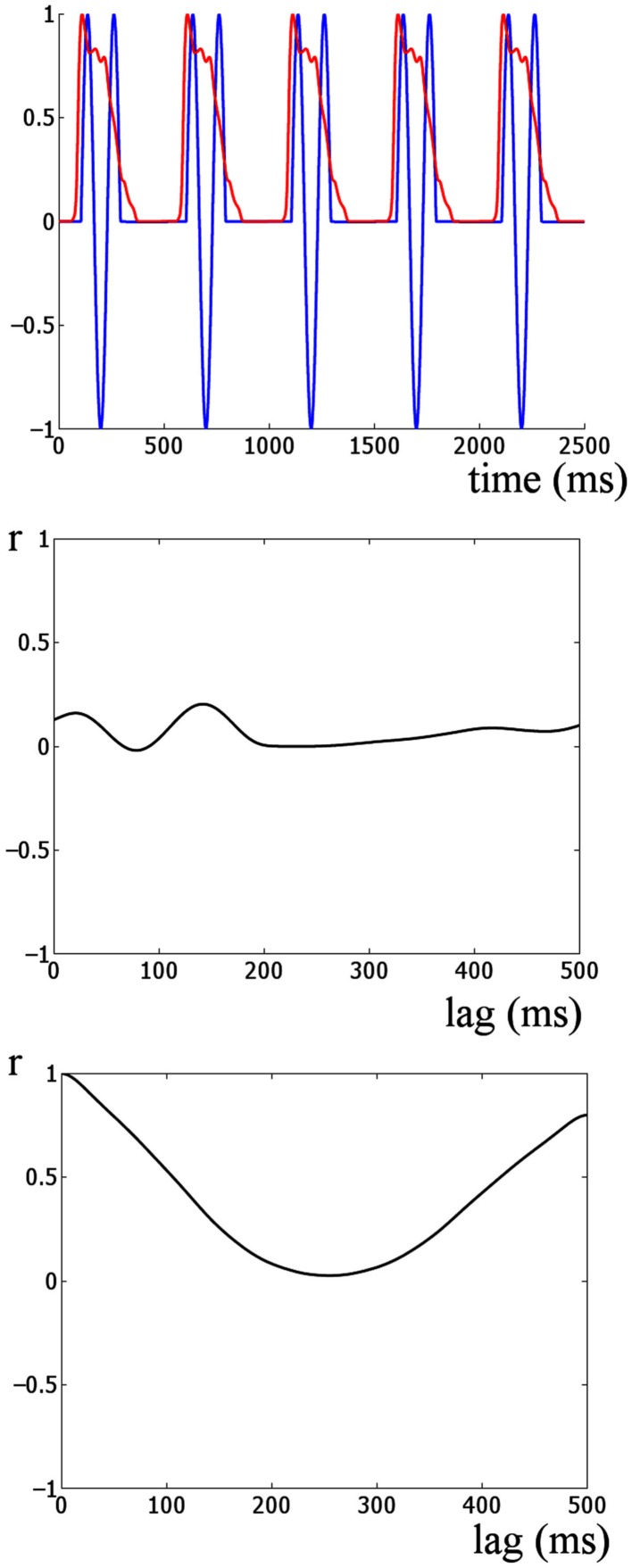
**Cross-correlation analysis of simulated onset ERP and stimulus envelope**. The upper panel shows the auditory stimulus envelope and an idealized onset response simulated by 1.5 cycles of an 8-Hz sine wave. This provides a model of how the responses would look if dominated by onset ERP activity. The middle panel shows the cross-correlation of the two signals shown in the upper panel. The lowest panel shows the autocorrelation of the auditory stimulus envelope. Two main differences are evident when the lowest panel is compared to the cross-correlations in the upper panel of Figure 4. Firstly, the strongest correlation for this simulation is much weaker than for the real auditory response (*r* = 0.2 versus *r* = 0.9). Further the shape of the cross-correlations is quite different. The peaks and troughs of the cross-correlations of the real data alternate between strong positive and negative correlation at approximately the stimulus rate. This is similar to the shape of the autocorrelation of the stimulus envelope (bottom panel). Thus overall the responses in the real data are indicative of an envelope following response and not indicative of a sequence of onset responses at the stimulation rate.

The relationship between the stimulus and the response was also tested via cross-correlation of the stimulus and the unfiltered responses (see Figure 4, this approach has also been employed previously, e.g., Abrams et al., 2008, 2009). Cross-correlation is not specific to frequency bands, but provides converging evidence regarding whether apparent entrainment is due to a stimulus-following response or rather is a result of onset responses occurring at the rate of stimulation. As can be seen in Figure 4, peak Pearsons *r*’s for the cross-correlations are clearly visible (the values are: A (at Cz), *r* = 0.90, at a 30-ms lag; V (at Oz), *r* = 0.78, at a 60-ms lag; AV (at Cz, cross-correlated with the auditory envelope, *r* = 0.72, at an 18-ms lag; and AV (at Oz, cross-correlated with the visual stimulus representation), *r* = 0.35, at a 10-ms lag; all *p*-values < 0.01). These correlations are much higher than would be obtained if the response was merely the result of onset responses occurring at the stimulation rate (see Figure 5, middle panel: *r*_max_ = 0.20 for idealized onset response). Furthermore, the topographies shown in Figure 4 show that the neural representation of the rhythmic stimulus is dominant in auditory and/or visual areas depending on the stimulation type. Both topographies for the AV condition (i.e., when cross-correlated with the auditory stimulus and the visual stimulus) are similar. This is to be expected given the strong correspondence between the auditory and visual stimulation.

Thus, the cross-correlation approach shows strong and significant stimulus representation in the responses, indicative of entrainment to the rhythmic stimulation.

Furthermore, computing an idealized onset response (Figure 5, lower panel) reveals that the shape of the cross-correlations of the idealized event-related potential (ERP) response are quite different from the shape of the real data (shown in the upper panel of Figure 4). The peaks and troughs of the cross-correlations of the real data alternate between strong positive and negative correlations at approximately the stimulus rate. However, the shape of the cross-correlation for the real data is similar to the shape of the autocorrelation of the stimulus envelope (Figure 5, bottom panel), further suggesting that the response and stimulus envelope are similar. Thus overall the data are indicative of an envelope following response rather than a sequence of onset responses at the stimulation rate.

### Behavioral performance and relations with entrainment in each condition

**Figure 6:**
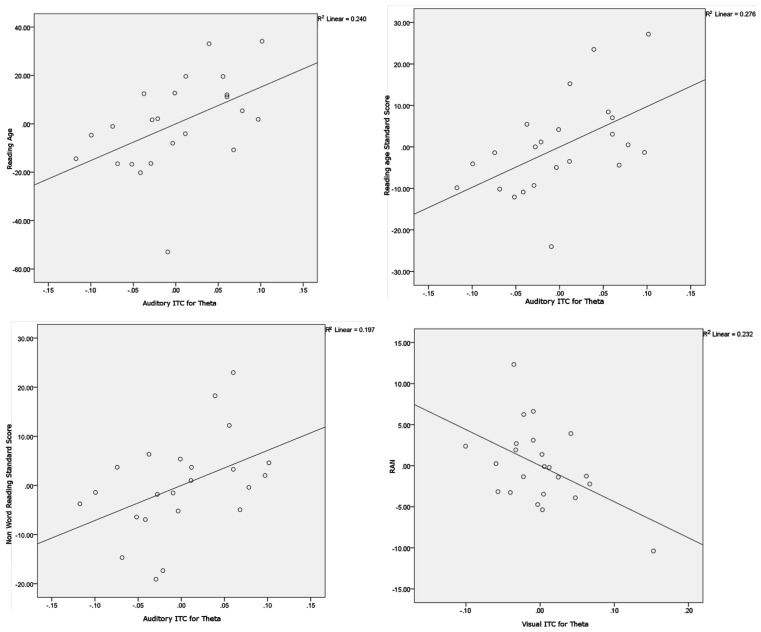
**Scatter plots and linear regression lines for the significant partial correlations**. Variables have been regressed onto the controlling variables before plotting. The plots show Auditory ITC for theta versus Reading age (upper left plot), Auditory ITC for theta versus Reading Standard Score (upper right plot), Auditory ITC for theta versus Non-word Reading Standard Score (lower left plot) and Visual ITC for theta versus Rapid Automatized Naming (RAN, lower right plot).

To explore whether individual differences in spoken and written language development were related to neural entrainment in the delta and theta bands, partial correlations controlling for age and IQ were computed for each condition. We chose the nine most strongly entrained electrodes in each case as a basis for these correlations, because the relevant topographical region changes with condition. The partial correlations are shown in Table 3. Scatter plots and linear regression lines for the significant correlations are shown in Figure 6. As noted earlier, on current multi-time resolution models of speech processing, auditory theta band entrainment should be most likely to show relations with language development in children. The correlations were indeed significant for the written language measures (*p*’s < 0.05), with the vocabulary measure approaching significance, *r* = 0.374, *p* = 0.095. No other correlations reached significance at the *p* < 0.05 level, with the exception of the rapid naming measure and visual theta ITC (*r* = −0.483, indicating that better entrainment goes with faster rapid naming performance). Hence in general the phonological processing measures (RAN, PSTM, and phoneme deletion) were not related to auditory entrainment, but the written language measures were.

**Table 3:** **Correlations between language, phonology, and neural entrainment**.

	Audit-ITC theta	Vis-ITC theta	AV-ITC theta	Aud-ITC delta	Vis-ITC delta	AV-ITC delta
BPVS	0.374+	−0.158	−0.020	−0.042	−0.016	−0.041
Read Age	0.489*	−0.082	−0.228	0.124	−0.163	0.014
Read SS	0.525*	0.026	−0.177	0.187	0.011	0.142
NonW SS	0.441*	0.221	−0.008	0.043	0.283	0.164
RAN	−0.186	−0.483*	−0.204	−0.390	−0.300	−0.347
PSTM	−0.146	−0.117	−0.402	−0.091	−0.009	−0.125
PhonDel	0.066	−0.325	−0.408	−0.056	0.047	−0.217

*p < 0.05.

### Effects of visual stimulation on auditory entrainment

#### Determination of entrainment ROI and phase locking in ROI

**Figure 7:**
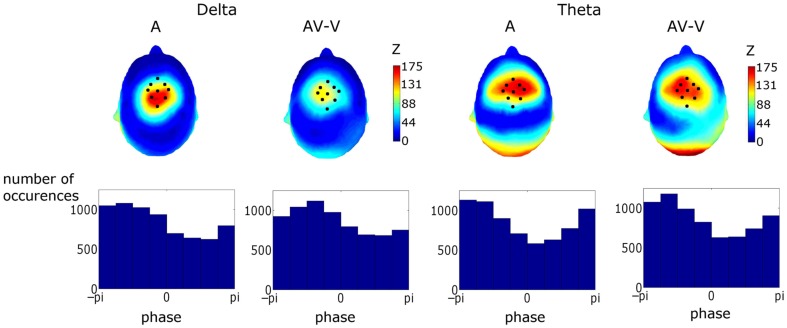
**Upper Panel: topographies of the Rayleigh statistic (*Z*) for phase at auditory stimulus onset (i.e., visual stimulus onset +68 ms)**. *Z* was set to zero where phase distributions were found not to differ from uniformity. The similarity of these topographies established a particular fronto-central region of interest coinciding with the area corresponding to the most significantly entrained region. Lower Panel: phase distributions for the conditions and frequency bands in the region of interest (ROI). Activity in the ROI showed significant entrainment, as tested using the Rayleigh statistic, in both frequency bands and for both response types. Tests on the preferred phase of entrainment showed that theta but not delta phase was affected by visual cues.

Figure 7 shows topographies of the Rayleigh statistic for the A and (AV–V) conditions. The fronto-central distribution in both conditions is indicative of entrainment in auditory cortical areas. Figure 7 also shows the phase distributions for delta and theta for the pooled activity in the ROI for both conditions. Rayleigh tests revealed significant entrainment in both conditions at both frequencies, as would be expected given the way in which the ROI was determined (A_δ_: *p* < 0.0001, *Z* = 150.75; AV–V_δ_: *p* < 0.0001, *Z* = 121.13; A_θ_: *p* < 0.0001, *Z* = 197.50; AV–V_θ_: *p* < 0.0001, *Z* = 177.40).

### Effects of natural visual cues on auditory oscillations

#### Entrainment (ITC)

We first compared the level of entrainment between conditions using ITC as the dependent measure. The ITC values were submitted to a 2 × 2 ANOVA with the factors of condition [A versus (AV–V)] and frequency band (δ versus θ). We found no main effect of condition (*p* > 0.05) or frequency band (*p* > 0.05), suggesting that the strength of phase locking in each condition and frequency band was similar. There was also no significant condition × frequency interaction (*p* > 0.05).

#### Power

Overall power was assessed in a similar manner to strength of entrainment. The induced power was submitted to a 2 × 2 ANOVA with the same factors of condition and frequency. Again, there was no main effect of condition (*p* > 0.05). There was a significant main effect of frequency, *F*(1,92) = 85.2, *p* < 0.001, η*p*^2^ = 0.492). *Post hoc* analysis (Tukeys) revealed that this effect was driven by power relationships in which δ > θ. This is to be expected, as EEG power generally decreases as frequency increases. Once again the condition × frequency interaction was not significant, suggesting that both frequencies were affected similarly by condition.

#### Preferred pre-stimulus phase

To assess whether the information from visual speech affected the phase of auditory entrainment, we tested for preferred phase differences between conditions using the Watson–Williams test for equal means of circular variables. We found no difference in the preferred phase in the delta band (*p* > 0.05). However, there was a significant difference in preferred phase in the theta band (*p* = 0.018). This can be seen in Figure 7, where the phase distribution in the (AV–V) theta condition can be compared to the A condition.

## Discussion

Here we assessed oscillatory entrainment in children to rhythmic speech using EEG, with repetition of the syllable “ba” presented either as auditory-alone (A), visual-alone (V), or auditory-visual speech (AV). Our paradigm also enabled us to assess whether cross-modal phase modulation of auditory entrainment by visual speech information could be measured in children, by comparing the A and (AV–V) responses. Stimuli were presented at an isochronous rhythmic rate of 2 Hz in each case. In the AV condition, the first visual articulatory movement preceded auditory onset by 68 ms. Our aims were to establish a viable paradigm for measuring oscillatory entrainment in children, and to investigate the role of congruent natural visual speech cues on auditory entrainment. As developmental language disorders like dyslexia and SLI may involve atypical oscillatory entrainment (Goswami, 2011; Lehongre et al., 2011; Hämäläinen et al., 2012), such a paradigm should enable future comparison of atypical auditory versus visual entrainment with atypical oscillatory function caused by different phase resetting of auditory oscillations by visual speech information.

We identified two frequency bands of interest (delta and theta) and assessed entrainment in each band in all three conditions. We found pre-stimulus phase entrainment in all three conditions, evidenced by the phase distribution differing significantly from a uniform random distribution, indicating a preferred concentration of phase for each type of speech. This is, to our knowledge, the first study to investigate and show neural entrainment in children. We also found that the strength of entrainment to speech at the theta rate was related to both spoken (*p* < 0.10) and written (*p*’s < 0.05) language ability. Our paradigm hence offers a potentially useful tool for the investigation of the developmental relationship between neural entrainment and speech processing and production. Theta activity is thought to be of critical importance for the parsing and encoding of syllable information in speech in healthy adults (Luo and Poeppel, 2007), and our data suggest a developmental link between theta entrainment and spoken and written language ability. In the current study, we found no differences in entrainment for auditory versus AV versus visual speech at the entrainment rate (2 Hz, delta), but we did find an entrainment difference between auditory and AV speech versus visual speech in the theta band. Further, in typically developing children, the strength of entrainment in the auditory condition compared to the (AV–V) response was equivalent. However, the preferred phase of auditory entrainment was altered in the presence of congruent visual stimulation. We interpret this as evidence of visual influence over auditory processing, with visual rhythmic stimulus streams modulating auditory oscillations to the optimal phase for auditory processing and audio-visual integration (Schroeder et al., 2008). These findings provide a baseline for future studies of entrainment in children with developmental language difficulties. Atypical auditory and possibly also visual entrainment has been hypothesized as one neural mechanism that may underpin the phonological impairments that characterize disorders of language development such as dyslexia and SLI (Goswami, 2011).

Of course, identifying the entrainment of endogenous oscillations (such as those discussed by Schroeder and Lakatos, 2009) as distinct from evoked stimulus-following oscillations [such as the steady-state response (SSR) e.g., Lehongre et al., 2011; Hämäläinen et al., 2012] is difficult with EEG. Both types of oscillation are forms of entrainment of neural activity and are likely to be heavily inter-related, as endogenous oscillations realign in such as way as to allow for optimal sensitivity when a stimulus is expected (Schroeder and Lakatos, 2009; Stefanics et al., 2010), and the extent of evoked stimulus-following responses will depend on whether important stimulus features fall in these optimally sensitive windows. The involvement of both endogenous and stimulus-following evoked activity is likely in the current data. Indeed the importance of phase-locked additive activity in speech processing has been recently highlighted (Howard and Poeppel, 2012). It is also mathematically possible that the peaks in the frequency spectra of the responses (shown in Figure 2) may be caused by onset ERP responses occurring in response to each stimulus, rather than to entrainment of neural activity at these frequencies *per se*. While evoked activity undoubtedly contributes to the power spectra, the fact that the frequency content of the responses closely mirrors that of the stimuli suggests that the EEG response is following the stimulus over time (as opposed to being dominated by an onset ERP response). This can be seen clearly in the differing importance of the theta activity in the auditory and visual responses, which mirror the differing importance of these frequencies in the stimulus (see upper panel Figure 2). In the lower panel in Figure 2, the establishment of an oscillation following the stimulus can be clearly seen. Converging evidence for this interpretation comes from the cross-correlation analysis, which shows a strong stimulus representation present in the response above and beyond that of onset responses.

It may also be possible that the phase entrainment seen here may be due to a concentration of phase resulting from the presence of an ERP component. However, phase values were extracted ∼435 ms post-stimulus onset. The prospect of an ERP component to an identical repeated stimulus having a large influence at this point is small. Furthermore, given the gradual offset of the stimuli (see Figure 1, top panel), it is also unlikely that an offset response (i.e., components related to a hard offset) played a role. Natural speech stimuli are not a sequence of discrete pulses, as each individual stimulus is extended considerably in time. Indeed the envelope of the stimulus sequence employed here resembles the envelope of natural speech rather than a train of discrete impulse stimuli. Thus it is likely that any evoked activity would be primarily the result of stimulus-following activity. The strength of the correlations between the stimulus and response observed here (Figure 4) is supportive of this interpretation.

The entrainment differences found for visual versus auditory and AV speech at the delta versus theta rates may be of conceptual interest. Schroeder et al. (2008) suggested that the mechanism for successful AV speech integration may involve the predictive modulation of auditory oscillations by visual speech cues. They identified both the delta and theta rates as likely to be particularly important for selective attention in speech processing (e.g., when following a single speaker during a cocktail party, see Zion Golumbic et al., 2012). In our paradigm, delta entrainment was not altered by visual speech information. However, each auditory stimulus predicted the next auditory stimulus with 100% temporal accuracy, hence it is likely that entrainment at the stimulus rate of delta was already in optimal or close to optimal phase in the auditory-alone condition. In the current investigation, the introduction of visual cues resulted in significant changes in preferred phase at the theta rate. Recently, Thorne et al. (2011) reported that the efficiency of auditory processing of non-speech tones was affected by visual cues that preceded the auditory stimulus. Both theta and alpha activity showed increased phase locking strength post-stimulus without an increase in power in auditory cortex when visual input preceded auditory input by 30–75 ms. In this study, the stimulation rate was 2.5 Hz, however only post-stimulus effects were calculated and so the data are not directly comparable with our data. Nevertheless, our findings are broadly in line with previous studies of cross-modal phase resetting involving adults.

Our data are also in line with a number of EEG and ECoG studies with adults which motivated the current study, all of which have shown anticipatory phase locking to visual stimuli or auditory tones presented in a rhythmic stream (e.g., Stefanics et al., 2010; Besle et al., 2011; Gomez-Ramirez et al., 2011). For neurotypical adults, Stefanics et al. (2010) found that the phase of the delta band oscillation just before a stimulus occurred was significantly related to the reaction time required to press a button to target tones. Hence entrainment of delta oscillations played a role in enhancing target detection. Besle et al. (2011) studied adult epileptic patients, and were able to take intracranial recordings during a paradigm involving interleaved auditory and visual rhythmic stimulus streams (following the paradigm devised for animals by Lakatos et al., 2008). They reported that the degree of entrainment depended on the predictability of the stream, and represented a reorganization of ongoing activity so that excitability was phase-shifted to align with the stimulation rate, in their case in the delta band (1.5 Hz). Gomez-Ramirez et al. (2011) also used intracranial recordings with patients, but used a presentation rate for rhythmic auditory and visual stimulus streams that was below the delta rate (0.67 Hz). They observed entrainment at twice the stimulation rate, suggesting that the brain can utilize oscillatory harmonics in selective attention tasks.

In summary, our data show that oscillatory entrainment to human speech can be measured successfully in children. In addition, we show that entrainment to auditory-alone and (AV–V) speech is similar in entrainment strength and in EEG power in 13-year-old typically developing children. Further, preferred phase was altered in the theta band by predictive visual speech information. Hence the functional role of visual speech on auditory oscillations can be demonstrated using our paradigm. These findings open the way to studying the developmental importance of oscillatory entrainment in disorders of language learning, both in terms of basic entrainment to auditory versus visual versus AV stimuli, and also in terms of the efficiency of cross-modal phase resetting mechanisms. The current paradigm could also inform possible interventions regarding auditory, visual, and/or audio-visual methods to facilitate entrainment.

## Conflict of Interest Statement

The authors declare that the research was conducted in the absence of any commercial or financial relationships that could be construed as a potential conflict of interest.
