# Erratum: Neural entrainment to rhythmically-presented auditory, visual and audio-visual speech in children

**DOI:** 10.3389/fpsyg.2013.00905

**Published:** 2013-12-03

**Authors:** Alan J. Power, Natasha Mead, Lisa Barnes, Usha Goswami

**Affiliations:** Department of Experimental Psychology, Centre for Neuroscience in Education, University of CambridgeCambridge, UK

**Keywords:** entrainment, audio-visual speech perception, rhythm, oscillations, language

Two inadvertent errors were discovered in Power et al. (2012). The first relates to Figure 3. This figure showed the histograms for one subject and not the whole group. A corrected Figure is included here. Rayleigh statistics carried out on these updated histograms reveal two differences in entrainment compared to the published results:

1. Theta activity at *Oz* in the visual condition, which was previously though not to be entrained, is in fact entrained.
2. Delta activity at *Oz* in the Audio-visual condition, which was previously thought to entrain to the stimulus, does not do so.

**Figure 3:**
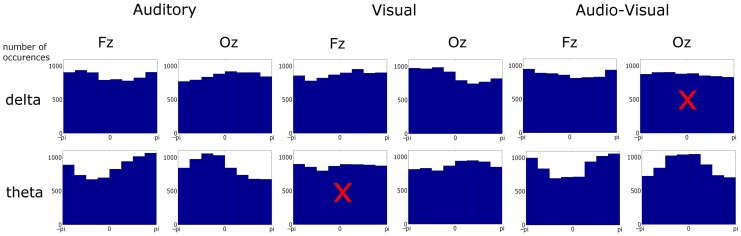
**Phase distribution at visual stimulus onset at representative frontal and occipital electrodes in each condition and frequency band over ~6900 observations in each plot**. Most distributions differed from uniformity when tested against the Rayleigh statistic at a critical *p*-value of 0.001. Distributions with a superimposed × did not result in significant entrainment.

Therefore, the published data should be adjusted as follows (differing results in bold italics):

A:
*Fz*_δ_: *Z* = 16.72, *p* < 0.0001. *Fz*_θ_: *Z* = 84.45, *p* < 0.0001
*Oz*_δ_: *Z* = 11.22, *p* < 0.0001. *Oz*_θ_: *Z* = 94.11, *p* < 0.0001
V:
*Fz*_δ_: *Z* = 11.66, *p* < 0.0001. *Fz*_θ_: *Z* = 2.07, *p* > 0.05
*Oz*_δ_: *Z* = 41.29, *p* < 0.0001. ***Oz*_θ_: *Z* = 14.91, *p* < 0.0001**
AV:
*Fz*_δ_: *Z* = 9.42, *p* < 0.001. *Fz*_θ_: *Z* = 92.85, *p* < 0.0001
***Oz*_δ_: *Z* = 2.45, *p* > 0.05**. *Oz*_θ_: *Z* = 90.62, *p* < 0.0001

The second error was observed when comparing Total Power in the A and (AV-V) conditions. ANOVA results should be as follows.

Repeated Measures ANOVA (frequency × condition) on Total Power:

- Frequency: *F*_(1, 22)_ = 261.62, *p* < 0.001, η*p*^2^ = 0.922
- Condition: *F*_(1, 22)_ = 13.76, *p* = 0.001, η*p*^2^ = 0.385
- Frequency × Condition: *F*_(1, 22)_ = 1.42, *p* > 0.05

Neither of these results effect the overall conclusions of the paper:

- Neural entrainment was demonstrated for all stream types, and individual differences in standardized measures of language processing were related to auditory entrainment at the theta rate.
- There was significant modulation of the preferred phase of auditory entrainment in the theta band when visual speech cues were present.

## References

[B1] Power A. J. Mead N. Barnes L. Goswami U. (2012). Neural entrainment to rhythmically presented auditory, visual, and audio-visual speech in children. Front. Psychol. 3:216 10.3389/fpsyg.2012.00216 22833726 PMC3400256

